# Medical Ethics

**Published:** 1874-11

**Authors:** 


### Our Exchanges.

*PHILADELPHIA MEDICAL TIMES—October*, 1874.

Medical Ethics.—A revolution in medical ethics, as we have stated before this, seems to us imminent, simply from the fact that there is an inevitable tendency for each individual to step a little in front of his fellows, and so to widen steadily the distance between the old and the present customs.

The splendid pecuniary success which Dr. Hammond claims to have attained, and of which he shows many tangible proofs, undoubtedly tempts men to follow his methods, and exerts an enormous influence—an influence which must be considered in at least some respects baneful by all those who revere the traditions of the fathers. We ourselves are tempted to believe that it would be fairer to the more scrupulous of the profession to abolish the Code of Ethics entirely than to let things drift as at present, and thus allow the use of well-tried business methods for success to those who are reckless of any professional public opinion which has not a dollars-and-cents value. As showing how the current is setting, we may mention the insertion, a few weeks since, by a "quiz organization" of a prominent medical college, of an advertisement in one of the evening penny papers. It was at a time when scarcely a student was in town, and the advertisement was in a paper that very few students *in* the city, certainly none *out* of it, see, and therefore could not possibly have availed in obtaining any students for the coming winter session. But then the names of the quizzers were in displayed type, each with his specialty set forth, and were scattered over the city among the very classes of the laity to be reached by such means. The kernel of the nut is not, we think, hard to discover. Even the defence of Dr. Hammond may serve as a sign-post of the times, if indeed it could with truth be granted that he has only done as do his professional brethren. How different in tone from Dr. Hammond's defence—which says in substance: "I allowed it; I furnished the wood-cuts to the newspapers; but the rest of you do the same thing"—is the recent indignant disavowal by Prof. Paget of any collusion with the publication of one of his lectures in the *Pictorial World!*—a disavowal in which he declares that he did all that he could do to prevent the publication, and adds, "I venture to hope that I am not thought likely to be guilty of encouraging the appearance of my name in newspapers."

